# Functional Potential of Red Dragon Fruit (*Hylocereus polyrhizus*) Juice By-Products as a Natural Feed Additive for Juvenile Red Seabream (*Pagrus major*): Implications for Antibiotic-Free Aquaculture

**DOI:** 10.3390/antibiotics14111096

**Published:** 2025-11-01

**Authors:** Hwa Yong Oh, Ki-Tae Kim, Tae Hoon Lee, Da Ye Kang, Do-Hyun Kwon, Young Wook Kim, Hee Sung Kim

**Affiliations:** 1 Department of Marine Biology and Aquaculture, Gyeongsang National University, Tongyeong 53064, Republic of Korea; oho1203@gnu.ac.kr (H.Y.O.); xogns2357@gnu.ac.kr (T.H.L.); honey3292@naver.com (D.Y.K.); qnf211@naver.com (D.-H.K.); kimmangkkong@naver.com (Y.W.K.); 2 Southeast Sea Fisheries Research Institute, National Institute of Fisheries Science, Tongyeong 53017, Republic of Korea; oysterkim@korea.kr

**Keywords:** red dragon fruit, *Pagrus major*, antibiotic alternatives, disease resistance, sustainable aquaculture

## Abstract

**Background:** The extensive use of antibiotics in aquaculture has raised serious concerns, emphasizing the need for sustainable and natural alternatives. This study evaluated the potential of red dragon fruit (*Hylocereus polyrhizus*) juice by-products (RJB) as a functional feed additive for juvenile red seabream (*Pagrus major*). **Materials and Methods:** The bioactive composition and antioxidant capacity of RJB were analyzed, and five experimental diets containing 0, 0.2, 0.4, 0.8, and 1% RJB were fed to fish for 56 days. **Results:** Growth performance, feed utilization, body composition, antioxidant enzyme activities, and lysozyme activity were evaluated. RJB contained substantial levels of phenolic and flavonoid compounds and exhibited strong radical-scavenging activity. Dietary inclusion of up to 1% RJB did not significantly affect growth, feed efficiency, or plasma biochemistry. However, fish fed the 1% RJB diet showed increased catalase and glutathione levels, significantly enhanced lysozyme activity, and improved survival following *Edwardsiella tarda* infection. **Conclusion:** These results demonstrate that RJB can be safely incorporated into marine fish diets to enhance antioxidant capacity and innate immune defense. The valorization of fruit-processing by-products such as RJB offers a promising strategy for developing antibiotic-free and sustainable aquaculture practices.

## 1. Introduction

Aquaculture is one of the fastest-growing food production sectors worldwide, driven by the increasing demand for aquatic products [1]. However, intensive high-density farming, which is commonly practiced to maximize productivity, imposes considerable stress on cultured species, leading to reduced growth, poor feed efficiency, and heightened disease susceptibility [2,3]. To mitigate these challenges, the widespread use of antibiotics and chemotherapeutic agents has been adopted in aquaculture. Although effective in the short term, their overuse has led to the emergence of antimicrobial resistance and environmental contamination, posing a global public health concern [4]. This situation highlights the urgent need for alternative, eco-friendly strategies that can promote fish health and immunity without relying on antibiotics.

Within this context, functional feed additives derived from agri-food by-products have attracted growing attention due to their bioactive properties and contribution to the “waste-to-wealth” and circular economy paradigms [5]. Fruit-processing residues are rich in polyphenols, flavonoids, and other phytochemicals with antioxidant and immunostimulatory potential [6,7]. Their inclusion in aquafeeds has been shown to improve growth performance, enhance innate immunity, and reduce disease susceptibility in various fish species [1,8,9]. Despite these promising outcomes, most available studies have focused on freshwater or omnivorous species, while the potential of such fruit by-products in marine carnivorous fish, which have distinct nutritional and metabolic requirements, remains largely unexplored.

Red dragon fruit (*Hylocereus polyrhizus*), a tropical cactus species widely cultivated in Southeast Asia and other regions, is valued for its vibrant color, high nutritional content, and health-promoting properties [10,11,12,13]. While primarily consumed as fresh fruit or juice, its processing generates substantial amounts of by-products—including peel, pulp residues, and seeds—which are typically discarded or used as low-value compost [14,15,16,17]. These by-products, however, are known to contain significant levels of betacyanin, flavonoids, and phenolic compounds with potent antioxidant activity [16]. Valorizing red dragon fruit juice by-products (RJB) as a functional feed ingredient not only addresses the issue of agricultural waste but also aligns with the principles of circular economy and sustainable aquaculture [18]. Despite the growing interest in plant-based feed additives, no studies have evaluated the use of RJB in marine carnivorous species.

Red seabream (*Pagrus major*) is a premium marine species extensively cultured in Korea and Japan, valued for its high market demand and taste [19]. In Korea, red seabream production accounted for approximately 8% of total marine aquaculture in 2024 [20]. Due to its economic importance and market demand, it plays a central role in regional aquaculture. However, due to its susceptibility to bacterial infections, especially those caused by *Edwardsiella tarda,* red seabream serves as an appropriate model species for evaluating the effectiveness of functional feed additives aimed at enhancing immunity and disease resistance [21,22,23]; yet, the potential of RJB for this species remains unexplored.

Therefore, this study aimed to (i) analyze the bioactive composition and antioxidant properties of RJB, and (ii) evaluate the effects of dietary RJB supplementation on growth performance, feed utilization, body composition, lysozyme activity, and disease resistance against *E. tarda* in juvenile red seabream. The findings contribute to the development of antibiotic-free, sustainable feeding strategies in marine aquaculture.

## 2. Results

### 2.1. Growth Performance and Feed Utilization

The growth performance and feed utilization of juvenile *P*. *major* after 56 days of feeding are summarized in Table 1. The survival rate ranged from 90.0% to 94.4% across all dietary treatments. There were no significant (*p* > 0.05) differences among treatments in growth performance [final body weight (FBW), weight gain (WG), specific growth rate (SGR), feed consumption (FC) and feed efficiency (FE)] or biometric indices (condition factor, hepatosomatic index, and viscerosomatic index).

### 2.2. Proximate Composition

The whole-body proximate composition of *P*. *major* fed experimental diets is presented in Table 2. Proximate composition (moisture, crude protein, crude lipid, and ash) did not differ significantly (*p* > 0.05) among treatments.

### 2.3. Plasma Biochemical Indices

Hematological and biochemical parameters are shown in Table 3. Plasma AST and ALT activities, as well as total cholesterol (T-CHO), total protein (TP), and glucose (GLU) concentrations, did not differ significantly (*p* > 0.05) among dietary groups.

### 2.4. Antioxidant Enzyme and Lysozyme Activities

Plasma antioxidant enzyme and lysozyme activities are presented in Table 4. The plasma antioxidant enzyme activities of *P*. *major* fed RJB-supplemented diets are presented in Table 4. No significant differences (*p* > 0.05) were observed in superoxide dismutase (SOD) activity among treatments, indicating that RJB inclusion up to 1% did not affect superoxide radical scavenging. However, catalase (CAT) and glutathione (GSH) levels exhibited significant differences among groups (*p* < 0.05). Both CAT activity and GSH concentration were significantly higher only in the RJB 1% group compared with the lower inclusion levels (*p* < 0.05). Fish fed the RJB1 diet (1% inclusion) showed significantly higher lysozyme activity compared with the control group (RJB0) (*p* < 0.05). However, no significant differences were observed among RJB0.2, RJB0.4, and RJB0.8 groups relative to the control (*p* > 0.05).

### 2.5. Resistance Against Edwardsiella Tarda Infection

**Figure 1:**
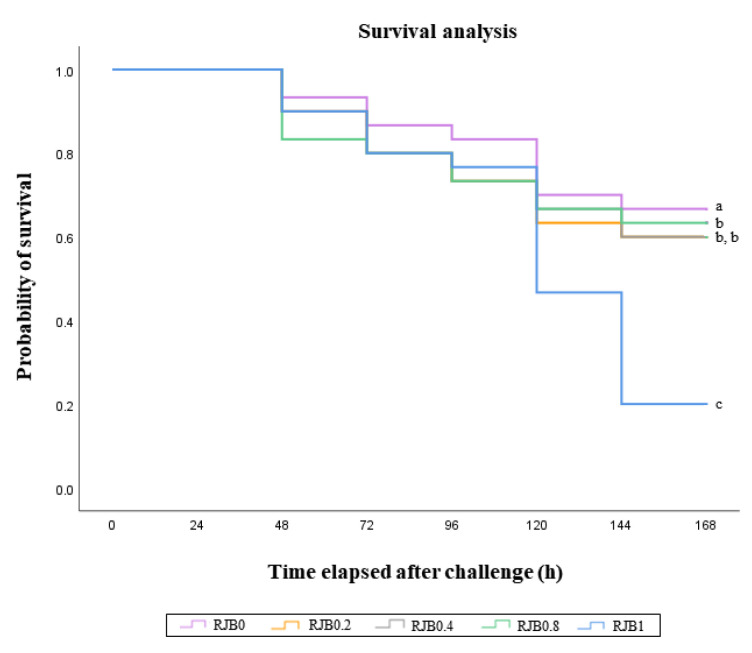
Survival of juvenile red seabream (*Pagrus major*) fed the experimental diets including red dragon fruit juice by-products (RJB) for 56 days, then artificially infected by *Edwardsiella tarda* (*p* < 0.001 for Log Rank and Wilcoxon tests). Different superscript letters (a, b and c) indicate significant differences among groups.

The cumulative survival of red seabream following *E*. *tarda* infection is presented in Figure 1. At day 7 post-infection, fish fed the RJB1 diet showed the highest survival rate among all groups, and Kaplan–Meier analysis further confirmed enhanced resistance to *E. tarda* (*p* < 0.001, log-rank and Wilcoxon tests).

## 3. Discussion

The RJB analyzed in this study were found to contain appreciable levels of flavonoids and phenolic compounds, which are widely recognized for their antioxidant and physiological regulatory functions. These phytochemicals act as free radical scavengers and metal chelators, thereby protecting cellular components from oxidative damage and maintaining redox balance [24]. The identified compounds are consistent with those previously reported in dragon fruit residues [16,25,26,27], all of which contribute to the overall antioxidant potential of plant-based feed additives. Previous studies have reported that red dragon fruit by-products, including peel and pulp residues, contain substantial amounts of betacyanins—water-soluble pigments with potent antioxidant properties [28]. Although the betacyanin content of RJB was not quantified in the present study, these pigments are known to exhibit strong radical-scavenging, anti-inflammatory, and cytoprotective activities. Therefore, it is likely that betacyanins, together with phenolic and flavonoid compounds, collectively contributed to the overall antioxidant potential of RJB. Also, these compounds are known to modulate endogenous antioxidant defense systems by enhancing the activities of antioxidant enzymes, and by upregulating transcription factors associated with oxidative stress response (e.g., Nrf2) [26,29].

The inclusion of RJB up to 1% in the diet of juvenile *P. major* did not significantly affect final body weight, weight gain, specific growth rate, or feed efficiency. These findings indicate that RJB supplementation neither enhanced nor impaired nutrient utilization or growth metabolism during the 56-day feeding trial. This neutral response may be attributed to the composition of RJB, which is rich in flavonoids and phenolic compounds—bioactive molecules that primarily act as metabolic modulators and antioxidants, rather than as direct nutrient sources [30,31]. Several plant-derived phenolics, such as catechin, rutin, and quercetin—identified in dragon fruit residues—are known to influence digestive enzyme activity and gut microbiota balance [32,33]. At moderate inclusion levels, these compounds may improve intestinal health and feed digestibility; however, their effects are often stabilizing rather than growth-promoting, particularly when fish are reared under non-stressful conditions [34]. The absence of significant growth improvement in this study suggests that RJB contributed to physiological homeostasis and oxidative stability, maintaining normal metabolic function without altering feed intake or conversion efficiency. Comparable results have been reported in *Dicentrarchus labrax* fed pineapple residues [35], *Seriola quinqueradiata* fed citrus peel powder [36], and *Oncorhynchus mykiss* fed grape pomace [37], where inclusion of fruit by-products-maintained growth performance without adverse effects. Conversely, excessive supplementation of fruit residues rich in fiber or tannins, such as pomegranate peel, has been shown to depress growth due to reduced digestibility [38]. The current findings imply that RJB at 1% provides a safe inclusion threshold, ensuring functional benefits from its antioxidant compounds while avoiding anti-nutritional effects associated with high polyphenol intake.

No significant differences were observed in the whole-body proximate composition of *P*. *major* fed diets containing up to 1% RJB. The similar levels of moisture, crude protein, lipid, and ash among treatments indicate that RJB supplementation did not alter nutrient deposition or body composition. This suggests that RJB inclusion did not interfere with protein synthesis, lipid metabolism, or water balance, which are closely linked to growth and feed efficiency. Comparable findings have been reported in *D. labrax* fed pineapple residues [35] and in *Sebastes schlegelii* fed blueberry by-products [39], where moderate inclusion of fruit-derived additives maintained normal body composition. The lack of change in proximate composition can be explained by the biochemical nature of RJB. Its major bioactive constituents—flavonoids and phenolic compounds—primarily exert regulatory rather than nutritive effects, functioning as antioxidants and metabolic stabilizers instead of as macronutrient contributors [40,41]. At the inclusion levels tested, these compounds likely helped maintain oxidative and metabolic homeostasis without modifying nutrient assimilation or tissue accretion. In general, polyphenol-rich plant additives improve physiological resilience and immune defense without necessarily altering proximate body composition under non-stressful rearing conditions [30,34].

Similarly, plasma biochemical parameters including AST, ALT, total cholesterol, glucose, and total protein remained unchanged across treatments, suggesting that dietary RJB had no detrimental effects on hepatic function or systemic metabolism. Stable AST and ALT levels indicate that hepatocellular integrity was preserved, while consistent total cholesterol and glucose levels reflect unaltered lipid and carbohydrate metabolism. These results demonstrate the metabolic safety of RJB and its compatibility with the nutritional physiology of *P. major*. The maintenance of normal plasma biochemical profiles is consistent with previous studies reporting that fruit by-product inclusion at moderate levels does not induce hepatic stress or metabolic imbalance in fish. For instance, no significant changes in AST, ALT, or cholesterol were observed in *Oreochromis niloticus* fed banana flower powder [42] or in carp fed lemon peel powder [43]. Although some studies have shown alterations in plasma metabolites when high doses of polyphenol-rich residues were used [44,45], the absence of such effects in the present study indicates that 1% RJB represents a physiologically safe supplementation level for *P. major*.

The antioxidant enzyme responses observed in this study indicate that dietary supplementation with RJB can enhance the oxidative defense capacity of juvenile *P. major*. Although SOD activity did not differ significantly among dietary treatments, CAT and GSH levels were significantly elevated in fish fed diets containing 1% RJB, demonstrating a dose-dependent improvement in enzymatic and non-enzymatic antioxidant activity. Since SOD catalyzes the dismutation of superoxide radicals into hydrogen peroxide, while CAT and GSH are responsible for its detoxification, the elevated CAT and GSH levels reflect an enhanced secondary antioxidant response and improved redox homeostasis [46]. The observed enhancement in CAT and GSH activities can be attributed to the flavonoid and phenolic compounds identified in RJB These compounds function both as direct ROS scavengers and as indirect regulators of endogenous antioxidant defenses by activating redox-sensitive transcription factors such as nuclear factor erythroid 2–related factor 2 (Nrf2), which enhances the expression of antioxidant enzymes and phase II detoxifying proteins, thereby promoting oxidative stress resilience in fish tissues [47]. Similar antioxidant upregulation has been reported in *O. niloticus* fed dragon fruit peel [29] and in *Clarias gariepinus* fed doum palm peel [48], indicating that phenolic-rich fruit by-products can stimulate enzymatic defense pathways without causing oxidative overload. The unchanged SOD activity observed here suggests that RJB did not induce oxidative stress but maintained a balanced oxidative environment while reinforcing hydrogen peroxide detoxification through CAT and GSH. Such a response pattern reflects preventive antioxidant modulation, a beneficial physiological adaptation where moderate polyphenol intake primes antioxidant defenses, enhancing readiness for subsequent immune challenges.

Consistent with this, fish fed the 1% RJB diet exhibited significantly higher serum lysozyme activity and improved survival following *E. tarda* infection. Lysozyme plays a pivotal role in the innate immune system by hydrolyzing bacterial peptidoglycans and promoting phagocytosis [49]. The enhanced lysozyme activity observed here suggests that the antioxidant protection conferred by RJB may have stabilized immune cell function and prevented oxidative suppression of innate immunity. In fish, oxidative stress can impair macrophage activity and lysozyme secretion [50]; thus, the strengthened antioxidant state in RJB-fed groups likely contributed to improved immune performance. Furthermore, fruit by-products rich in phenolic compounds have been shown to simultaneously enhance antioxidant and immune responses in fish, as evidenced by increased lysozyme activity in *Labeo rohita* fed lemon peel powder [51], *Lates calcarifer* fed passion fruit peel [52], and *C*. *gariepinus* fed doum palm peel [48], with these immunostimulatory effects attributed to the synergistic action of polyphenols, flavonoids, and polysaccharides that promote macrophage activity, complement function, and cytokine signaling. The higher post-challenge survival observed in the RJB1 group further supports the link between antioxidant enhancement and immune protection. *E. tarda* infection is known to induce oxidative stress in fish [53], leading to lipid peroxidation and immune suppression. The antioxidant compounds in RJB likely mitigated these effects by maintaining cellular redox equilibrium and supporting the activity of immune effector enzymes. Similar associations between antioxidant status and disease resistance have also been demonstrated in *O. niloticus* fed prickly pear peel [54] and *C. gariepinus* supplemented with doum palm peel [48].

Taken together, the present findings demonstrate that dietary supplementation with RJB, a fruit by-product–derived ingredient, can enhance antioxidant defense and innate immune capacity in *P. major*. However, these effects were primarily evaluated at the biochemical and physiological levels. To fully elucidate the functional potential of RJB and similar fruit by-products as sustainable feed additives, further molecular studies are warranted to identify the signaling pathways and gene regulatory networks involved in their antioxidant and immunomodulatory actions. Future investigations focusing on the activation of transcription factors such as Nrf2 and NF-κB, and the expression of downstream antioxidant- and cytokine-related genes, will provide mechanistic insight into how fruit by-products modulate redox balance and immune function. Such molecular-level understanding would not only clarify the biological basis of these responses but also support the valorization of agro-industrial by-products as eco-friendly and functional ingredients in aquafeed formulations.

## 4. Materials and Methods

### 4.1. Preparation of Red Dragon Fruit Juice By-Products (RJB)

Red dragon fruit was purchased from World Market Co. (Busan, Republic of Korea; origin: Bình Thuận Province, Vietnam). After washing, the fruit was processed using a juicer (H-300L-DBFC03, Hurom Co. Ltd., Seoul, Republic of Korea) to separate juice and by-products. The RJB were dried at 40 °C for 48 h using a drying oven (KED-M07D1; Kiturami Co. Ltd., Seoul, Republic of Korea), ground into powder, and stored at −20 °C until diet preparation.

### 4.2. Functional Compounds and Antioxidant Capacity of RJB

The total phenolic and flavonoid contents of RJB were 23.96 mg/100 g and 16.49 mg/g, respectively (Table 5). Antioxidant assays revealed that RJB exhibited strong radical-scavenging capacity in both DPPH and ABTS tests, with inhibitory activity increasing in a dose-dependent manner.

### 4.3. Determination of Functional Compounds and Antioxidant Activity of RJB

Total phenolic content was determined using the Folin–Ciocalteu method [55]. Absorbance was measured at 700 nm, and gallic acid (Sigma-Aldrich, St. Louis, MO, USA) was used as a standard. Total flavonoid content was analyzed following the procedure of [54]. Absorbance was measured at 415 nm, and quercetin (Sigma-Aldrich, St. Louis, MO, USA) was used as a standard. DPPH radical scavenging activity was measured using the method of [56]. A methanolic DPPH solution (150 μM) was mixed with 80 μL of sample or ascorbic acid (positive control), incubated at room temperature for 10 min, and absorbance was read at 525 nm. ABTS radical scavenging activity was analyzed following the method of [57]. ABTS radical solution was generated by incubating 7 mM ABTS with 2.4 mM potassium persulfate in the dark for 16 h. The working solution was diluted to an absorbance of 1.5 at 414 nm, and mixed with samples or ascorbic acid before absorbance measurement at 414 nm.

### 4.4. Preparation of Experimental Diets

Five experimental diets were formulated (Table 6). The control diet (RJB0) contained no RJB, while the experimental diets included 0.2%, 0.4%, 0.8%, and 1% RJB (RJB0.2, RJB0.4, RJB0.8, and RJB1, respectively). Wheat flour in the control diet was partially replaced with RJB in the experimental diets. Fish meal and defatted soybean meal were used as the main protein sources, fish oil and soybean oil as lipid sources, and wheat flour as the carbohydrate source. The ingredients were thoroughly mixed, pelleted into 3 mm diameter pellets using a laboratory pelletizer, and dried at 24 °C for 48 h. The diets were stored at −35 °C until use. Fish were fed three times daily (09:00, 13:00, and 17:00) to apparent satiation throughout the 56-day trial.

### 4.5. Experimental Fish and Rearing Conditions

Juvenile *P. major* were obtained from a private fish farm located in Pohang, Gyeongsangbuk-do, Republic of Korea. The fish were transported to the Marine Bio-Education and Research Center of Gyeongsang National University (Tongyeong, Gyeongsangnam-do, Republic of Korea), where they were acclimated for two weeks. During acclimation, fish were fed a commercial sinking diet (Chunha Feed Co., Haman, Republic of Korea; crude protein: 52%, crude lipid: 10%) twice daily to apparent satiation. Prior to the feeding trial, fish with an average initial weight of 6.2 ± 0.00 g were fasted for 24 h and randomly distributed into fifteen 50 L rectangular flow-through plastic tanks (30 fish per tank, three replicates per treatment). The tanks were supplied with continuous aeration, and the water flow rate was maintained at 2.7 L/min. Water temperature, salinity, and dissolved oxygen were monitored daily using a YSI Pro Plus multiparameter (YSI Inc., Yellow Springs, OH, USA) and recorded as 20.6 ± 0.12 °C, 30.5 ± 0.06 psu, and 6.0 ± 0.11 mg/L, respectively. The feeding trial was conducted for 56 days.

### 4.6. Growth Performance and Biometric Measurements

At the end of the feeding trial, fish were fasted for 24 h and anesthetized with tricaine methanesulfonate (MS-222, 100 ppm; Sigma-Aldrich, St. Louis, MO, USA). Final body weight and survival rate were recorded for each tank. Ten fish per tank were randomly sampled for growth performance indices including WG, SGR, FE, and CF HSI, and VSI were calculated by weighing the liver and viscera, respectively.

### 4.7. Blood Sampling

Blood samples were collected from the caudal vein of 10 fish per tank using both heparinized and non-heparinized syringes after anesthesia with MS-222 (100 ppm). Plasma and serum were separated by centrifugation (8000 rpm, 10 min) and stored at −80 °C until analysis.

### 4.8. Proximate Composition Analysis

The proximate composition of each experimental diet was analyzed in triplicate subsamples. Whole-body samples (10 fish per tank) were homogenized and analyzed in triplicate for proximate composition, including moisture, crude protein, crude lipid, and ash, following the procedures described by [58]. Crude protein was determined using a KjelROC Analyzer (OPSIS Liquid LINE, Furulund, Sweden), crude lipid by ether extraction (Soxtec™ system, FOSS, Hillerød, Denmark), moisture by oven-drying at 105 °C for 24 h, and ash by combustion in a muffle furnace at 550 °C for 4 h.

### 4.9. Plasma Biochemical Analysis

ALT, AST, T-CHO, TP, and GLU were measured using an automated biochemical analyzer (FUJI Dri-Chem NX500i, Fujifilm, Tokyo, Japan).

### 4.10. Antioxidant Response

To evaluate antioxidant status, plasma levels of SOD, CAT, and GSH were measured using commercial assay kits (Cayman Chemical, Ann Arbor, MI, USA). All analyses were carried out according to the manufacturers’ protocols. Absorbance values were read using a MULTISKAN GO microplate reader (Thermo Scientific, Vantaa, Finland).

### 4.11. Lysozyme Activity

Serum lysozyme activity was determined following [59]. Briefly, *Micrococcus lysodeikticus* suspension (0.4 mg/mL in phosphate buffer, pH 6.2) was mixed with serum samples in 96-well plates, and the decrease in absorbance at 600 nm was recorded every 5 min for 30 min using a spectrophotometer (Thermo Fisher Scientific, Tewksbury, MA, USA).

### 4.12. Bacterial Challenge Test

After the feeding trial, the *E. tarda* strain FP5060 was obtained from the Korean Collection of Aquatic Microorganisms (National Institute of Fisheries Science, Busan, Republic of Korea). Ten fish per tank were intraperitoneally injected with 0.1 mL of bacterial suspension (1.6 × 10^5^ CFU/mL). Mortality was monitored for 10 days, and dead fish were immediately removed. The mean water temperature during the challenge was 24.4 ± 1.52 °C.

### 4.13. Statistical Analysis

All data are presented as mean ± standard error (SE). Statistical analyses were performed using SPSS version 27.0 (IBM, Chicago, IL, USA). One-way ANOVA followed by Tukey’s HSD test was used to determine significant differences among treatments (*p* < 0.05). Kaplan–Meier survival analysis, log-rank, and Wilcoxon tests were conducted to analyze survival data following bacterial challenge.

## 5. Conclusions

This study demonstrated that dietary inclusion of RJB up to 1% had no adverse effects on the growth performance, feed utilization, or proximate composition of juvenile *P. major*. Supplementation with 1% RJB significantly enhanced serum lysozyme activity and improved post-challenge survival against *E. tarda*, indicating its potential as a natural immunostimulant. These findings suggest that RJB, a low-value by-product of juice processing, can be feasibly utilized as a functional feed additive in marine aquaculture. However, its role as an antibiotic alternative remains a working hypothesis that requires further verification. Future research should focus on elucidating the molecular mechanisms underlying its immunomodulatory effects, determining optimal inclusion levels, and evaluating its long-term efficacy under commercial farming conditions.

## Figures and Tables

**Table 1:** Growth performance and feed utilization of juvenile red seabream (*Pagrus major*) after 56 days of feeding with the experimental diets.

Experimental Diets	RJB0	RJB0.2	RJB0.4	RJB0.8	RJB1	*p*-Value
IBW ^1^ (g/fish)	6.2 ± 0.01 ^a^	6.2 ± 0.00 ^a^	6.2 ± 0.00 ^a^	6.2 ± 0.00 ^a^	6.2 ± 0.00 ^a^	-
FBW ^2^ (g/fish)	30.6 ± 0.32 ^a^	31.3 ± 1.03 ^a^	28.7 ± 2.09 ^a^	26.7 ± 1.32 ^a^	27.4 ± 2.73 ^a^	0.310
WG ^3^ (g/fish)	24.4 ± 0.32 ^a^	25.1 ± 1.03 ^a^	22.5 ± 2.09 ^a^	20.5 ± 1.33 ^a^	21.1 ± 2.73 ^a^	0.316
SGR ^4^ (%/day)	3.39 ± 0.023 ^a^	3.43 ± 0.069 ^a^	3.24 ± 0.149 ^a^	3.09 ± 0.106 ^a^	3.12 ± 0.218 ^a^	0.325
Survival (%)	92.2 ± 1.11 ^a^	90.0 ± 1.92 ^a^	92.2 ± 1.11 ^a^	94.4 ± 2.94 ^a^	93.3 ± 3.33 ^a^	0.720
FC ^5^ (g/fish)	25.5 ± 0.89 ^a^	23.4 ± 0.96 ^a^	23.4 ± 2.21 ^a^	22.0 ± 1.44 ^a^	22.9 ± 1.14 ^a^	0.524
FE ^6^	0.96 ± 0.042 ^a^	1.09 ± 0.073 ^a^	0.97 ± 0.016 ^a^	0.95 ± 0.013 ^a^	0.93 ± 0.076 ^a^	0.301
CF ^7^	1.90 ± 0.101 ^a^	1.93 ± 0.051 ^a^	1.93 ± 0.030 ^a^	1.89 ± 0.043 ^a^	1.79 ± 0.073 ^a^	0.551
VSI ^8^	2.23 ± 0.078 ^a^	2.13 ± 0.066 ^a^	2.04 ± 0.114 ^a^	2.04 ± 0.052 ^a^	2.08 ± 0.117 ^a^	0.557
HIS ^9^	1.88 ± 0.243 ^a^	1.63 ± 0.054 ^a^	1.95 ± 0.123 ^a^	1.73 ± 0.104 ^a^	1.61 ± 0.051 ^a^	0.339

Values are presented as mean ± SE of triplicate groups, and different superscript letter in the same row indicate significant differences (*p* < 0.05). ^1^ IBW, initial body weight; ^2^ FBW, final body weight; ^3^ Weight gain (WG, g/fish) = final body weight-initial body weight; ^4^ Specific growth rate (SGR, %/day) = (In final weight of fish-In initial weight of fish)/days of feeding trial × 100; ^5^ Feed consumption (FC, g/fish) = dry feed consumed; ^6^ Feed efficiency (FE) = weight gain of fish/feed consumed; ^7^ Condition factor (CF) = fish weight/total length^3^; ^8^ Viscerosomatic index (VSI, %) = 100 × (visceral weight/body weight); ^9^ Hepatosomatic index (HSI, %) = 100 × (hepatopancreas weight/body weight).

**Table 2:** Proximate composition of red seabream (*Pagrus major*) fed experimental diets for 56 days.

Experimental Diets	RJB0	RJB0.2	RJB0.4	RJB0.8	RJB1	*p*-Value
Moisture (%)	70.1 ± 0.05 ^a^	70.0 ± 0.07 ^a^	70.4 ± 0.16 ^a^	70.1 ± 0.20 ^a^	70.4 ± 0.14 ^a^	0.281
Crude protein (%)	17.8 ± 0.02 ^a^	17.6 ± 0.06 ^a^	17.6 ± 0.07 ^a^	17.8 ± 0.07 ^a^	17.6 ± 0.04 ^a^	0.114
Crude lipid (%)	6.7 ± 0.16 ^a^	6.5 ± 0.04 ^a^	6.4 ± 0.08 ^a^	6.4 ± 0.12 ^a^	6.7 ± 0.05 ^a^	0.106
Ash (%)	4.3 ± 0.11 ^a^	4.6 ± 0.08 ^a^	4.3 ± 0.10 ^a^	4.6 ± 0.13 ^a^	4.4 ± 0.16 ^a^	0.388

Values are presented as mean ±SE of triplicate groups, and different superscript letter in the same row indicate significant differences (*p* < 0.05).

**Table 3:** Blood chemistry of red seabream (*Pagrus major*) fed experimental diets for 56 days.

Experimental Diets	RJB0	RJB0.2	RJB0.4	RJB0.8	RJB1	*p*-Value
AST ^1^ (U/L)	44.3 ± 5.84 ^a^	31.7 ± 3.76 ^a^	64.3 ± 13.45 ^a^	33.3 ± 4.63 ^a^	44.0 ± 15.82 ^a^	0.227
ALT ^2^ (U/L)	8.3 ± 0.88 ^a^	6.7 ± 0.33 ^a^	9.0 ± 1.15 ^a^	6.0 ± 0.58 ^a^	9.3 ± 2.33 ^a^	0.321
T-CHO ^3^ (mg/dL)	239.7 ± 15.06 ^a^	251.0 ± 3.46 ^a^	242.0 ± 10.07 ^a^	236.0 ± 6.81 ^a^	236.3 ± 20.83 ^a^	0.917
GLU ^4^ (mg/dL)	75.0 ± 15.01 ^a^	100.0 ± 4.04 ^a^	100.0 ± 13.45 ^a^	78.7 ± 8.21 ^a^	86.7 ± 5.49 ^a^	0.330
TP ^5^ (g/dL)	4.2 ± 0.17 ^a^	4.0 ± 0.13 ^a^	3.9 ± 0.06 ^a^	3.7 ± 0.09 ^a^	3.7 ± 0.20 ^a^	0.122

Values are presented as mean ±SE of triplicate groups, and different superscript letter in the same row indicate significant differences (*p* < 0.05). ^1^ AST, aspartate aminotransferase; ^2^ ALT, alanine aminotransferase; ^3^ T-CHO, total cholesterol; ^4^ GLU, glucose; ^5^ TP, total protein.

**Table 4:** Plasma antioxidant enzyme and serum lysozyme activities of red seabream (*Pagrus major*) fed experimental diets for 56 days.

Experimental Diets	RJB0	RJB0.2	RJB0.4	RJB0.8	RJB1	*p*-Value
SOD ^1^ (U/mL)	2.3 ± 0.10 ^a^	2.4 ± 0.11 ^a^	2.4 ± 0.21 ^a^	2.5 ± 0.08 ^a^	2.7 ± 0.05 ^a^	0.278
CAT ^2^ (nmol/min/mL)	258.7 ± 6.39 ^a^	269.2 ± 7.49 ^a^	279.0 ± 8.36 ^ab^	309.0 ± 17.10 ^ab^	336.0 ± 14.29 ^b^	0.009
GSH ^3^ (µM)	16.7 ± 0.43 ^a^	16.5 ± 0.80 ^a^	19.3 ± 0.95 ^ab^	20.6 ± 0.60 ^ab^	23.0 ± 1.99 ^b^	0.005
Lysozyme (U/mL)	0.061 ± 0.007 ^a^	0.068 ± 0.004 ^ab^	0.089 ± 0.013 ^ab^	0.095 ± 0.004 ^ab^	0.123 ± 0.024 ^b^	0.048

Values are presented as mean ±SE of triplicate groups, and different superscript letters in the same row indicate significant differences (*p* < 0.05). ^1^ SOD, superoxide dismutase; ^2^ CAT, catalase; ^3^ GSH, glutathione.

**Table 5:** Total phenolics and total flavonoids contents, and radical scavenging activities of the red dragon fruit (*Hylocereus polyrhizus*) juice by-products (RJB).

	RJB Composition				
Chemical compounds					
Total phenolics (gallic acid mg/100 g)	23.96 ± 9.78				
Total flavonoids (quercetin mg/g)	16.49 ± 8.34				
Radical scavenging activities					
Concentration (μg/mL)	2000	1000	500	250	IC_50_
DPPH ^1^ (%)	49.43	27.95	16.36	6.68	4.8
ABTS ^2^ (%)	36.15	21.78	12.12	5.32	5.0

^1^ DPPH, 1,1-diphenyl-2-picrylhydrazyl; ^2^ ABTS, 2,2′-azinobis-(3-ethylbenzothiazoline-6-sulfonate).

**Table 6:** Formulation and proximate composition of the experimental diets (DM basis, %) containing different concentration of red dragon fruit (*Hylocereus polyrhizus*) juice by-products (RJB).

	Experimental Diets				
	RJB0	RJB0.2	RJB0.4	RJB0.8	RJB1
Jackmackerel meal	60	60	60	60	60
Dehulled soybean meal	11	11	11	11	11
Wheat flour	19.5	19.3	19.1	18.7	18.2
RJB ^1^	0	0.2	0.4	0.8	1
Fish oil	3.5	3.5	3.5	3.5	3.5
Soybean oil	3.5	3.5	3.5	3.5	3.5
Vitamin premix ^2^	1	1	1	1	1
Mineral premix ^3^	1	1	1	1	1
Choline	0.5	0.5	0.5	0.5	0.5
Proximate composition (%)					
Dry matter	93.7	93.6	92.9	93.6	92.7
Crude protein	51.7	50.8	52.1	51.7	52.2
Crude lipid	13.1	12.9	13.6	13.5	14.1
Ash	12.2	12.6	13.1	12.8	12.6

^1^ RJB (red dragon fruit juice by-products) was purchased from the local store. ^2^ Vitamin premix contained the following amount which were diluted in cellulose (g/kg mix): L-ascorbic acid, 121.2; DL-α-tocopheryl acetate, 18.8; thiamin hydrochloride, 2.7; riboflavin, 9.1; pyridoxine hydrochloride, 1.8; niacin, 36.4; Ca-D-pantothenate, 12.7; myo-inositol, 181.8; D-biotin, 0.27; folic acid, 0.68; p-aminobenzoic acid, 18.2; menadione, 1.8; retinyl acetate, 0.73; cholecalciferol, 0.003; cyanocobalamin, 0.003. ^3^ Mineral premix contained the following ingredients (g/kg mix): MgSO_4_·7H_2_O, 80.0; NaH_2_PO_4_·2H_2_O, 370.0; KCl, 130.0; ferric citrate, 40.0; ZnSO_4_·7H_2_O, 20.0; Ca-lactate, 356.5; CuCl, 0.2; AlCl_3_·6H_2_O, 0.15; KI, 0.15; Na_2_Se_2_O_3_, 0.01; MnSO_4_·H_2_O, 2.0; CoCl_2_·6H_2_O, 1.0.

## Data Availability

Data available on request.
